# Main Applications and Recent Research Progresses of Additive Manufacturing in Dentistry

**DOI:** 10.1155/2022/5530188

**Published:** 2022-02-24

**Authors:** Gan Huang, Libo Wu, Jie Hu, Xiongming Zhou, Fei He, Li Wan, Shu-Ting Pan

**Affiliations:** ^1^Department of Stomatology, The First Affiliated Hospital of Nanchang University, Nanchang, 330006 Jiangxi, China; ^2^Department of Stomatology, The Third Hospital of Nanchang, Nanchang, 330000 Jiangxi, China

## Abstract

In recent ten years, with the fast development of digital and engineering manufacturing technology, additive manufacturing has already been more and more widely used in the field of dentistry, from the first personalized surgical guides to the latest personalized restoration crowns and root implants. In particular, the bioprinting of teeth and tissue is of great potential to realize organ regeneration and finally improve the life quality. In this review paper, we firstly presented the workflow of additive manufacturing technology. Then, we summarized the main applications and recent research progresses of additive manufacturing in dentistry. Lastly, we sketched out some challenges and future directions of additive manufacturing technology in dentistry.

## 1. Introduction

Additive manufacturing (AM) is commonly called three-dimensional (3D) printing technology. There are other more synonyms of AM in the research literature, such as additive fabrication, additive processes, additive techniques, additive layer manufacturing, layer manufacturing, freeform fabrication, rapid manufacturing, direct digital manufacturing, and rapid prototyping [1, 2]. The American Society for Testing and Materials (ASTM) has defined AM as “a process of joining materials to make objects from 3D model data, usually layer upon layer, as opposed to subtractive manufacturing methodologies” [2]. The International Organization for Standardization (ISO), another globally recognized leader in the arena of international standards, declares that the definition of AM shall be in accordance with the ASTM F2792 standard [3, 4]. AM technology has been developed for over 40 years and has reached a maturity level that can be converted into commercial applications in many industries including automotive, aerospace, consumer products, and biomedical engineering [5, 6]. AM technology is based on the data of a 3D mathematical model and continuous layered printing technologies [7]. Unlike a subtractive manufacturing process, AM can directly produce complex three-dimensional structures with improved manufacturing accuracy, simplified production process, economized materials and human resources, shortened production time, and improved production efficiency and can be utilized in precision medicine to achieve personalized needs [7–10]. In the field of dentistry, AM technology has been more and more widely used and researched concerning maxillofacial surgery, denture implantation, prosthodontics, and orthodontics, from the production of the personalized surgical guides to the fabrication of maxillofacial alternatives, dental implant, and the manufacture of internal crowns, skeletons for implants and dental restorations, etc. [11–15]. The application of AM technology has led to the development of dentistry from traditional pure empirical methods to digitization and precision [16]. The widespread use of cone-beam CT (CBCT) can rebuild three-dimensional maxillofacial and dental anatomy which significantly improves the quality of diagnosis and treatment and expedites the development of AM in dentistry [17]. Besides, bioprinting using AM which incorporates active ingredients such as cells, matrix, and growth factors has shown amazing development potential in the field of tooth, jawbone, and periodontal tissue regeneration. This review surveys the data, materials, and methods in AM technology that are helping to enable improvement in precision dentistry. Application of these additive manufactured platforms will also be described to provide a historical perspective while focusing on the state of the art in dentistry.

## 2. Historical Evolution of AM Technology

**Figure 1:**
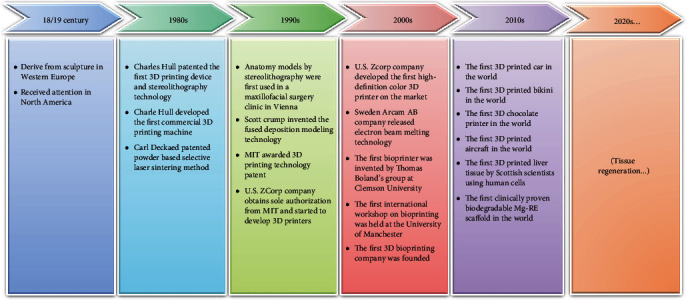
Historical evolution of additive manufacturing.

The germ of additive manufacturing technology originated from the sculpture art of Western Europe in the 18th century and has received attention in North America in the 19th century. With the development of computer and network technology in the 20th century, 3D printing technology was really born. And the evolution of AM technology has tremendously accelerated at present (Figure 1). In 1980s, American engineer Charles Hull patented the first 3D printing device and technology named as stereolithography [18, 19]; in 1986, Charles Hull developed the first commercial 3D printing machine, the SLA-1; in 1989, Carl Deckard patented the powder-based selective laser sintering (SLS) method where the powdered material is melted and refused by a laser beam [20].

In 1991, human anatomy models produced by stereolithography were first applied in a maxillofacial surgery clinic in Vienna; in 1992, Scott Crump invented the fused deposition modeling (FDM) method to print a 3D structure [21]; in 1993, Massachusetts Institute of Technology (MIT) obtained the patent for 3D printing technology; in 1995, the United States ZCorp company got the sole authorization from MIT and started to develop 3D printers.

In 2003, Sweden Arcam AB company first introduced commercial electron beam melting (EBM) equipment in the world; in September 2004, the first international workshop on bioprinting was held at the University of Manchester [22]; in 2005, the first high-definition color 3D printer Spectrum Z510 on the market was successfully developed by ZCorp; in 2007, the first 3D bioprinting company Organovo was founded [23]; in November 2010, the world 's first printed car Urbee came out; in June 2011, the world's first 3D-printed bikini was released; in July 2011, British researchers developed the world's first 3D chocolate printer; in August 2011, engineers from the University of Southampton developed the world's first 3D-printed aircraft; in November 2012, Scottish scientists used human cells to print artificial liver tissue with a 3D printer for the first time [24]; in 2016, Germany Biotronik company realized the first clinically proven biodegradable magnesium-based scaffold for coronary artery lesion in the world [25]. With the advent of new materials and improvement of AM technology, tissue engineering will be developed rapidly.

## 3. Workflow of AM Technology

**Figure 2:**
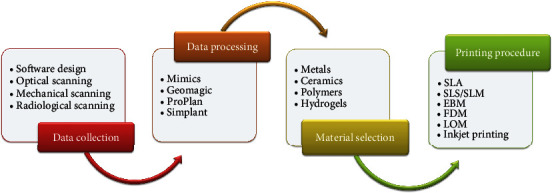
Workflow of additive manufacturing.

Just as illustrated in Figure 2, the main workflow of AM technology comprises data collection, data processing, material selection, and the final printing procedure. Data collection and processing are the fundamental steps and often used to establish a digital model via various types of CAD software. And the digital model should be solidified by the optimal material and corresponding manufacturing procedure. On the other hand, the selection of a certain material and printing procedure is dependent on the application or research purpose.

### 3.1. Data Collection

The collection of 3D data is an important step in model making, as low-resolution images can lead to discrepancy between the generated model and actual anatomy [26]. At present, there are four common methods: software design, optical scanning, mechanical scanning, and radiological scanning. Models designed using design software do not have to be constrained to the size of real objects and are convenient for calculation, analysis, modification, and editing [27]. Optical scanning commonly applies three-dimensional laser scanning, projection raster measurement, moiré fringe method, or stereo photography. It has a higher scanning rate and better accuracy, but the complex shape will make the scanning a blind spot, and errors will occur in the scanned data [28]. Handheld optical scanning showed a low geometric assessment for imaging a complete denture form [29]. With the increase in the freedom degree of the mechanical probe, the scanning blind area will be reduced [30]. With the development of computer tomography (CT) and nuclear magnetic resonance (MRI) technology, radiological diagnosis has become less invasive and more accurate [31]. The high-resolution 3D image data can be obtained within seconds, making radiological scanning an ideal method for 3D data acquisition [32]. CT data reconstructed from slice thicknesses of 0.5-1 mm are be suitable for maxillofacial surgery models [33]. Still and all, the potential risks of ionizing radiation remain a concern [34]. Despite the fact that MRI offers a nonionizing alternative to CT, simple thresholding-based segmentation is unsuitable due to the overlapping pixel values of bone, soft tissue, and air [35]. To solve this problem, Eley et al. developed a semi- and fully automated segmentation algorithm utilizing short echo time (ZTE) for the craniofacial skeleton. This technique is transferrable to a routinely used non-bone-specific sequence (FIESTA-C) imaging which offers enhanced resolution and reduced acquisition time [36, 37].

### 3.2. Data Processing

A special high-performance computer is generally used to process the obtained 3D data into the 3D reconstruction software. Segmentation is a commonly used step in medical applications to isolate the area of interest within the data set. The scanned data in terms of DICOM (Digital Imaging and Communications in Medicine) format is imported into the software such as Mimics, Geomagic, ProPlan, and Simplant. There are some differences between the features of software packages particularly in the fine and thin areas of osseous structures; it is essential to understand the features of software package for the intended purpose [38]. Generally, these types of software can read the DICOM data and rebuild the 3D model [39, 40]. The threshold tool allows one to retain or remove areas of interest according to density values of tissue types. Another tool for segmenting is seed-based region growing which employs a starting point or seed and sets voxel density parameters. Additional voxels which meet the defined density criteria are then added to the seed [41]. After segmentation, volumetric data are converting into 3D triangular mesh surfaces with the help of automated surface rendering tools. The quantity of triangle “fragments” is positively related to the model accuracy and graph smoothness [26]. The process of segmentation and mesh generation may bring about significant inaccuracies between the original DICOM data and the final 3D generated model. To ensure a true anatomical presentation, it is critical to compare the region of interest with the original DICOM data after every major step [42]. Then, the reconstructed data is saved as the STL (Surface Tessellation Language) format. Finally, the STL data can be recognized and processed by the 3D printer [16].

### 3.3. Material Selection

#### 3.3.1. Metals

Many metals and metal alloys are available, but only a few are safe to implant into people. For biomedical applications, metal products are required to own good mechanical properties, biocompatibility, and corrosion resistance properties [43]. Biocompatibility of a metallic material guarantees the desired function of the material without inducing harmful local or systemic effects on the surrounding tissue. Currently, metallic materials including stainless steel, cobalt chromium alloy, and titanium and titanium alloy are biologically compatible and can be used for dental applications [44]. In the 1920s, stainless steels (SS) are widely used due to the properties of corrosion resistance, enhanced strength, and bargain price [45]. SS 316 and 316 with low carbon content (L) are the most commonly used stainless steel alloys in the field of maxillofacial reconstruction [46]. These alloys contain chromium, molybdenum, and nickel yielding corrosion resistance [47]. However, these stainless steel alloys are not suitable for manufacturing permanent implants due to their low mechanical properties [48]. In the 1930s, cobalt chromium (Co-Cr) alloys, possessing better biocompatibility and wear resistance and higher hardness than SS 316, have been applied successfully for orthodontics and prosthodontics in dentistry [49].

Titanium (Ti) and titanium alloy materials have the advantages of small density, high accuracy and large mechanical strength, and good biocompatibility. Porous titanium and titanium alloys have been successfully used in dental application since the end of the 1960s [50]. Moreover, they are regarded as ideal 3D-printing metal materials [51] and have been widely used, especially in the reconstruction of oral and maxillofacial [52] and manufacturing of a dental implant [53]. Due to some defects of pure titanium, for example, the strength of pure titanium is not as great as that of titanium alloys, and the elastic modulus of pure titanium is higher than that of bone tissue, which can easily lead to incompatible mechanical stress between titanium implants and bone. Many researchers have tried various ways to improve the performance of pure titanium, such as adding a coating on its surface or oxidized pure titanium surface [54–56]. Nonstochastic geometry titanium with a periodic repetition of lattice structures shows better mechanical properties and enhanced osseointegration [57]. Traini et al. formed a gradient titanium-6 aluminium-4 vanadium (Ti-6Al-4V) titanium alloy porous dental implant, which has more optimized physical and chemical properties. The tensile strength, section shrinkage, and elongation are up to AMs4999 (standards on 3D-printed titanium alloys issued by ASTM) [58]. The elastic modulus of the sintered titanium core is similar to that of machined titanium, while the elastic modulus of the porous layer on the surface of this laser sintered titanium alloy implant is reduced thus similar to that of cortical bone. It is beneficial to the long-term stability of the implant [58]. After heat treatment, the mechanical strength of Ti-6Al-4V can be increased up to 50% without affecting Young's modulus [59]. Not only can the classical Ti-6Al-4V alloy be printed but also the second generation of titanium alloys, such as Ti-12Mo-6Zr-2Fe, Ti-15Mo-5Zr-3Al, To-13Nb-13Zr, and Ti-15Sn-4Nb-2Ta-0.2Pd, has been developed for biomedical printing [60]. These titanium alloys containing Nb, Ta, and Zr (*β* stabilizing elements) have low moduli of elasticity and spare toxicity of the aluminium or vanadium [61].

The metallic implants can be fabricated using AM technologies which are powder bed fusion processes. Selective laser sintering (SLS), selective laser melting (SLM), and electron beam melting (EBM) are the most established methods that rely on local fusion of metal particles to form a solid material [62]. Metal powders used in AM need to be spherical and have a stringent particle size distribution to achieve good packing effect [44]. Compared with casting and milling techniques, SLS-fabricated CoCrMo dental alloys had better mechanical properties and less dissolution of metal ions [63]. An engineered porous structure of metallic alloys by AM technology helps to match the stiffness of the implant with that of the natural bone [64]. Thus, the stress shielding effect can be addressed. Moreover, the porosity also facilitates bone cell growth, creating solid osseointegration between the bone and the implant [65].

The properties of the printed metal materials vary across the printing methods. For example, Ti-6Al-4V printed via SLS exhibits an *α* martensitic microstructure, while Ti-6Al-4V printed via EBM shows a needle-like *α* + *β* Widmanstatten microstructure [66]. The mechanical properties of SLS-printed Ti-6Al-4V are anisotropic, different from the isotropic mechanical properties of EBM-printed Ti-6Al-4V. Besides, the residual stress and porosity of SLS-printed Ti-6Al-4V are larger than those of EBM-printed Ti-6Al-4V [66]. One step worth mentioning is the post-AM heat and surface treatments. By this, AM porous metal materials can obtain improved microstructure and mechanical properties [67, 68]. Hot isostatic pressing, combining high temperature with high pressure level, can be applied to close the cavernous defects generated by the AM process [67, 69]. Sand blasting and chemical etching are used to remove unmolten powder thus to improve the surface properties of AM porous implants before using them *in vivo* [67, 69].

#### 3.3.2. Ceramics

In dentistry, ceramic materials are required to have aesthetics and biocompatibility as well as low density, high strength, high hardness, high temperature resistance, corrosion resistance, and good physical and chemical properties. Ceramics such as zirconia and alumina are currently used in artificial dental bridges and crowns. Zirconia can be also used in dental implants which display comparable osseointegration with titanium [70]. When zirconia ceramics are fabricated by cutting technology, a lot of materials will be cut off resulting in waste and high price of the all-ceramic crown. This craft process may also cause internal cracks by cutting forces in the denture. The AM technology in fabricating zirconia ceramic dentures can reach more than 90% of material utilization, lower the price, and reduce environmental pollution [71]. Noteworthy, 3D-printed zirconia can achieve the bioimitability such as hardness by printing special internal structures [72]. The AM procedure for fabricating zirconia is mainly based on the laser sintering method, but there are some problems such as low density and forming efficiency and surface cracks [73]. Stereolithographic ceramic manufacturing has good surface quality and controllability of structural accuracy [74, 75] and quickly becomes a research hotspot. At present, there are still some problems in the AM process of zirconia materials, such as large internal stress, cracking after sintering, and volume shrinkage, which may affect its mechanical properties and clinical suitability. Ceramic materials and their fabricating technology still need further investigation.

#### 3.3.3. Polymers

Polymer materials have become the basic mature printing material in the field of 3D printing. In dentistry, polylactic acid (PLA), polycaprolactone (PCL), and polyetheretherketone (PEEK) are relatively common 3D printing materials. PLA is an environmentally friendly material with good biodegradability. It can be completely degraded by microorganisms in nature under specific conditions and eventually generate carbon dioxide and water, which will not cause environmental pollution and is very beneficial to environmental protection. It also has translucency and gloss texture, making it an ideal material for 3D printing in the field of dentistry. A 3D-printed scaffold using a blend of PCL and gelatin has superior mechanical flexibility and softness which could be suitable for soft tissue engineering such as rhinoplasty. Human adipose-derived stem cell was cultured on this scaffold and showed increased cartilage differentiation and tissue formation [76].

Polyetheretherketone (PEEK) is a thermoplastic polymer. PEEK material has a similar modulus of elasticity to human bones, and the stress of the skull after repair is complete; X-ray transmission performance is good, no metal artifacts are generated, it does not affect medical images, and it is convenient to detect postoperative recovery; currently, PEEK is used to manufacture denture parts. The Oxford Performance Materials company used PEKK to print craniofacial bone repair patch which has been approved by the US Food and Drug Administration (FDA).

#### 3.3.4. Hydrogels

The hydrogels, typical and commonly used materials in bioprinting, have a 3D network structure with hydrophilic polymer chains, and the water content is 90% to 99%, which contributes to effective oxygen and substance exchange. In the past few decades, hydrogels have achieved unprecedented development in the construction of tissue engineering scaffolds and drug carrier due to their high biocompatibility, low immunogenicity, and adjustable physical and chemical properties [77, 78]. The hydrogel multimer system can provide a good matrix for cell transplantation and differentiation, endogenous regeneration, bioremediation, wound healing, and continuous drug delivery, while its three-dimensional network system can simulate the microstructure of the original extracellular matrix and provide living ecological conditions for cell survival [79, 80]. However, at present, the hydrogel prepared by 3D drawing organisms has low hardness, which may lead to structural collapse or the complexity of shape restriction. To optimize the structure and function of hydrogel, many efforts have been made. Gelatin methacryloyl- (GelMA-) based hydrogel can undergo photopolymerization of methacryloyl substituents to generate covalently crosslinked hydrogels which are very suitable for manufacturing biological constructs with unique patterns and morphologies [81, 82]. Hyaluronic acid (HA), a water-soluble glycosaminoglycan, has been formulated into hydrogels [83]. Methacrylates and acrylates can modify HA via amide, carboxylate, and dydroxyl functionalities [84]. Methacrylated HA has been applied in tissue engineering scaffolds as well as the bioprinting of cell-laden scaffolds [82, 85]. Moreover, to improve the reliability and printing resolution, hydrogel can be deposited into a granular support bath with small, uniform, and spherical microparticles [86, 87].

### 3.4. Printing Procedure

**Figure 3:**
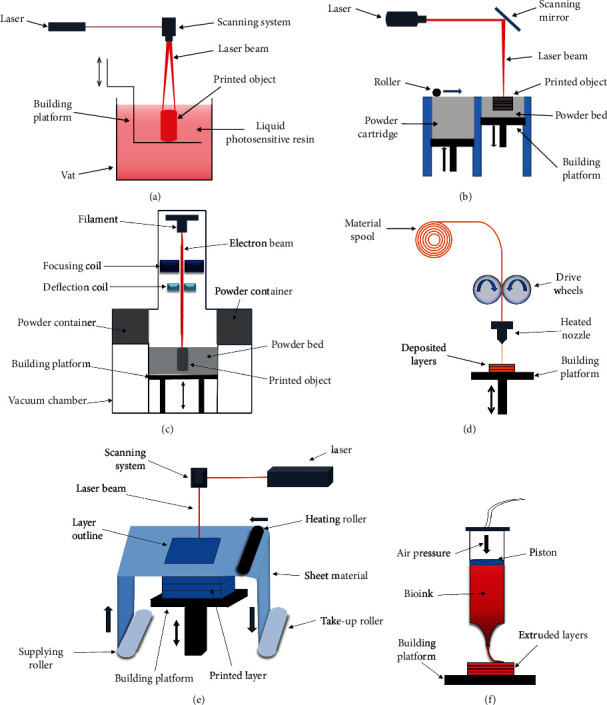
The schematic diagrams of various AM procedures including SLA (a), laser-based printing (b), EBM (c), FDM (d), LOM (e), and inkjet printing (f).

The printing procedure applied in dentistry mainly comprises stereolithography (SLA), laser-based printing, electron beam melting (EBM), fused deposition modeling (FDM), laminated object manufacturing (LOM), and inkjet printing (IJP). The merits and schematic diagrams of these AM technologies are shown in Table 1 and Figure 3, respectively.

#### 3.4.1. Stereolithography

Stereolithography (SLA) uses a photopolymer to cure by ultraviolet or visible light irradiation. The material of the SLA process is photosensitive polymers which can rapidly polymerize under the irradiation of specific wavelength and intensity ultraviolet light, thereby transferring from liquid to solid with high accuracy. SLA technology has the characteristics of rapid fabrication time, high precision, and stable performance which can be applied to form complex architectures [88]. Due to the limitation of the material, this printing method needs a supporting structure. The manufactured products are generally brittle and easily broken and require a strict storage [89]. The ideal SLA system depends on the careful calibration of light type and intensity, photoinitiator/photoabsorber sensitivities and concentration, and material concentration and reactivity [90]. This SLA printing technology is widely used in restoration of defected teeth, complete denture base, resin crown, dowel crown, implant guide, and tooth root canal model.

#### 3.4.2. Laser-Based Printing

Selective laser sintering (SLS) and selective laser melting (SLM) technology can be vastly used in processing small particles of metal, polymer, and ceramic materials, including titanium and titanium alloy powder, cobalt chrome, stainless steel, nylon powder, and elastomers. The advantages are high good mechanical property, good accuracy, and high material utilization rate. The disadvantages are easy to produce spherification which affects the product quality [91]. For metal and metal alloys, SLS is also described as direct laser metal sintering (DLMS) or direct laser metal formation (DLMF) which is commonly used method in the maxillofacial and denture implant.

SLM uses laser to melt metal powder which is a physical-chemical metallurgical change. This technology overcomes the overcomplicated SLS process. The manufactured products have better compactness and higher performance but are prone to spheroidization which requires continuous improvement and optimization of this procedure [92]. SLS and SLM are mainly used in implant guide, implant, and alternative of large jawbone defects.

#### 3.4.3. Electron Beam Melting

Electron beam melting (EBM) is a new type of direct manufacturing technology for metal parts developed in recent years. It is a process of selectively melting metal powders by high-energy electron beams and depositing them layer by layer until the required metal parts are manufactured. EBM technology can easily, quickly, and accurately complete the manufacture of extremely complex morphological parts and can especially form a complex three-dimensional interconnected pore structure, which provides structural conditions for orthopedic implants to induce bone cell ingrowth. Implants have unique advantages and become an important way to meet individual needs [92].

#### 3.4.4. Fused Deposition Modeling

Fused deposition modeling (FDM) is to eject molten thermoplastic material such as polycarbonate or eutectic metal powder and immediately solidify it. The material filaments are heated in the hot melt nozzle through the transmission mechanism, then extruded and solidified through the nozzle, and finally formed layer upon layer. The molding speed is fast and relatively accurate, without expensive laser sintering equipment, and the price is relatively low. This kind of materials is mostly used in oral implant prostheses [93]. Polylactic acid composite materials (such as hydroxyapatite/polylactic acid) can be selected to induce the adhesion of osteoblasts in the alveolar socket to the prosthesis [94]. Compared with traditional oral prostheses, this technique significantly saves the treatment cost, shortens the operation time, and improves the strength of the prosthesis

#### 3.4.5. Laminated Object Manufacturing

Laminated object manufacturing (LOM) is to use glue to glue paper or plastic film together and then use laser to shape. The thin material of LOM adheres layer by layer under the action of hot melt adhesive. The advantage of this technology is that the price of raw materials is relatively low, and the accuracy is acceptable, but the surface of the manufactured product is relatively rough with obvious ladder patterns and easy to crack, so this technology is usually used to make jawbone and denture models and formulate comprehensive and effective surgical plan models [95].

#### 3.4.6. Inkjet Printing

Inkjet printing (IJP) can be used to print living cells and biological materials to construct a three-dimensional biological scaffold containing different tissues, even living organs. The mixture of hydrogels and cells are distributed into series of droplets using thermally driven nozzle. By layers of printing, three-dimensional structures containing cells can be formed. IJP technology has many advantages such as high resolution, reproducibility, inexpensiveness, and ease of use. Thermally driven IJP is of fast printing speed, and the printing nozzles generate bubbles through local resistance heating, squeezing the liquid in the nozzle to obtain droplets. However, its performance in droplet direction, uniformity, and size control is not satisfactory, and the thermal stress, nozzle clogging, cell exposure, and other problems generated during the ejection process are often detrimental to printing influences. And the shear force generated by inkjet 3D printing is easy to cause loss of cells. Therefore, improving the survival rate of inkjet 3D printing cells and optimizing the printing process still face challenges [91].

## 4. Applications and Recent Research Progresses of AM Technology in Dentistry

**Figure 4:**
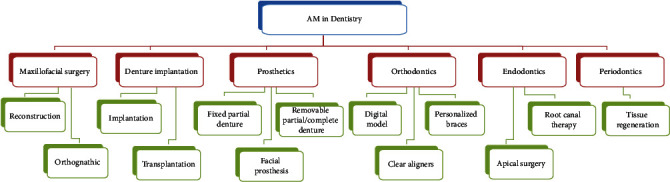
Main applications of additive manufacturing in dentistry.

AM technology is widely used in the biomedical field due to its high precision and personalized characteristics [110]. FDA has cleared some 3D-printed devices for clinical use including orthopedics devices, surgical guides, and dental bridges [111, 112]. In dentistry, the main applications and research directions of 3D printing include the following: three-dimensional stereoscopic image software combined with prototype models for anatomy teaching guidance [113, 114], preoperative planning and drills [115], prognosis analysis and judgment [116], personalized treatment equipment, assistive devices and implants including personalized orthodontic brackets and accessories [117, 118], restorations [119], trays [96], implants and surgical guides [120, 121], and bioactive materials combined with living cells and growth factors to print tissues and organs with bioactive functions [122, 123] (Figure 4). Nevertheless, the bioprinting is still at the stage of printing biological scaffolds and needed further exploitation to mimic complex structure and function of natural tissues in the human body [124].

### 4.1. AM Technology in Maxillofacial Surgery (Table 2)

#### 4.1.1. Reconstruction

**Figure 5:**
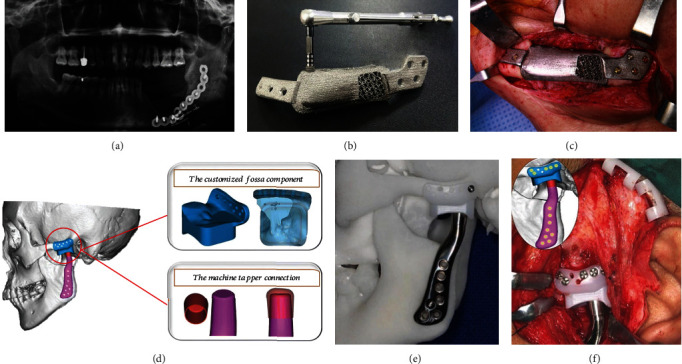
Panoramic radiograph demonstrated huge defect of the left mandible due to osteoradionecrosis (a); a titanium mandibular substitute with premounted dental implant fixtures manufactured by SLM (b) was employed to restore the bony defect (c). Reprinted from [130]. Customized temporomandibular joint (TMJ) prosthesis comprised of the fossa, condylar head, and mandibular handle components (d); the novel TMJ prosthesis can precisely match the Chinese patient's TMJ anatomy (e); the lateral view of a fixed TMJ prosthesis during a surgical procedure (f). Reprinted from [131].

Maxillofacial trauma and tumors can cause maxillofacial fractures and bone defects, and restoring the normal anatomy of the maxillofacial region and language function is of great significance to improve the quality of life. In the 1990s, scholars began to try to apply 3D printing technology to the preoperative evaluation of maxillofacial surgery, formulating surgical plans and simulations. Compared with traditional milling models, the stereolithographic 3D models have much higher accuracy [125]. And the application of stereolithographic technology has improved the diagnostic accuracy by 29.60% and the operation accuracy by 36.23% and shortened the operation time by 17.63% [126]. In 2012, the Institute of Biomedical Research at Hasselt University in Belgium used 3D printing technology to make a pair of titanium alloy mandibles for an 83-year-old patient. The patient recovered language and swallowing function one day after surgery. Melville et al. and Takano et al. used the CAD/CAM technology to create a model for fibular flap transplantation for partial maxilla resection and used the model to create a device for guiding the position of the mandible excision and a titanium plate for fibular flap fixation. This procedure shortened surgery time and improved safety, function, and esthetic outcomes [127, 128]. Haider et al. demonstrated a feasible in-house virtual surgical plan (VSP) and 3D-printed cutting guides in maxillofacial reconstruction. 19 patients with maxillofacial tumors undergoing microvascular bone reconstruction were managed by this technique. The average time for VSP and fabrication of cutting guides was 158 minutes. The average cost was $18.01 Canadian dollars [129]. This in-house VSP and 3D printing are operable for the surgeon and can benefit patients. Park et al. [130] have recently introduced a novel 3D-printed Ti implant to restore huge mandibular defect caused by osteoradionecrosis (Figure 5(a)). In order to rehabilitate mastication, three dental implant fixtures were installed in this mandibular implant during the surgery (Figures 5(b) and 5(c)). Moreover, customized temporomandibular joint (TMJ) prosthesis has been employed for the treatment of end-stage TMJ osteoarthrosis (Figures 5(d)–5(f)). Twelve patients recruited in this clinic trial reported an average of 90.7% decrease in pain, 70.8% improvement in mandible function, 79.9% improvement in diet, and 32.8% increase in maximal interincisal opening (MIO) postsurgery 1 year. The anatomical structure of this TMJ prosthesis can match that of the individual patient [131].

#### 4.1.2. Orthognathic Surgery

Orthognathic surgery is commonly applied to treat skeletal malocclusion which severely affects the occlusion function and the facial features of patients. In the 21st century, AM technology is more and more widely used in orthognathic surgery measurement [132]. There are many kinds of 3D-printed surgical guiding templates such as repositioning guides, osteotomy, and predrilling guides.

**Figure 6:**
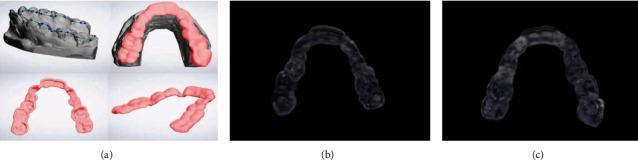
The surgical splint for orthodontic treatment can be designed via CAD software (a); thereafter, SLA technology and biocompatible photosensitive resin were employed to print this splint with sophisticated features (b, c). Reprinted from [138].

Dumrongwongsiri et al. used the 3D-printed Le Fort I spacers to guide maxilla-mandibular repositioning for 12 patients with facial asymmetry and malocclusion. The average time for preoperative simulation, design, and printing of these spacers was 2.5 hours. The average cost was 40 dollars per space. All the patients were satisfied with postoperative facial symmetry and occlusion [133]. Wang et al. applied a 3D-printed mandible model and surgical templates to simultaneously perform orthognathic and mandibular contour osteoplasty for treating mandibular protrusion [134]. Shaheen et al. and Heufelder et al. proposed an optimized protocol using 3D planning-printing for bimaxillary orthognathic surgery. 95% of 3D-printed splints were clinically accepted [135]. The median deviation of the maxilla position was 0.39 mm between the preoperative plan and surgical result [136]. Li et al. evaluated a customized orthognathic surgical guide for splint-less bimaxillary surgery. The largest root-mean square deviations for maxillary dental arch, mandibular arch, mandibular body, and proximal segments were all below 1.1 mm and 2.82°. The median surgical time was 160 minutes. All patients achieved good final occlusion [137]. The personalized orthognathic surgical splint is accurate and effective for the sake of patients and surgeons (Figure 6) [138].

### 4.2. AM Technology in Denture Implantation (Table 3)

#### 4.2.1. Implantation

**Figure 7:**
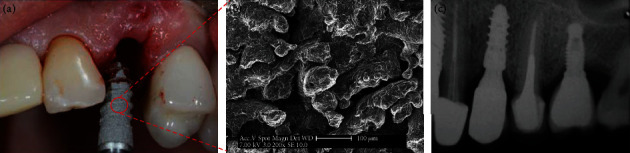
A commercialized 3D-printed Ti6Al4V implant (Tixos®, Leader Implants, Italy) was going to be inserted in the socket (a); the surface of this printed implant consisted of tremendous grooves with 14.6 to 152.5 *μ*m in width and 21.4 to 102.4 *μ*m in depth (b); the printed dental implant possessed satisfactory osteointegration after 3 years of functional loading due to its rough surface (c). Reprinted from [121].

Denture implantation is a widely accepted and prevailing treatment modality to replace lost teeth. Titanium alloys are commonly used materials in dental implants due to their excellent chemical stability and biocompatibility [139]. However, the stress shielding effect caused by the mismatch of elastic modulus between the bone and titanium implant may lead to bone loss adjacent to implants [140]. Recently, AM has gained great attention for fabricating complex porous implants with improved mechanical preformation to reduce the stress shielding effect and enhance the osseointegration between bones and the implant [58, 141]. Depending on the variation of porosity and pore size, the elastic modulus and compressive strength of the printed titanium or titanium alloy implants can coincide with those of human cortical and cancellous bone [142]. Besides, AM technology is suitable for patients whose bone mass is inadequate and implants need to avoid important anatomical structures. Compared with the commercial NobelActive^TM^ implant, the 3D-printed porous Ti6Al4V dental implant has higher biomedical parameters and better osseointegration [143]. Customized root form dental implant fabricated by the method of EBM technology was reported to possess superior rough and porous surface texture which facilitated the implant stabilization and osseointegration [144]. Tunchel et al. [121] launched a multicenter study to check the survival and success rates of AM titanium dental implants after three-year loading (Figure 7). The results with 94.5% survival rate and 94.3% success rate display a prosperous clinical option for the restoration of single tooth gaps using AM titanium implants [121]. Mangano et al. and Figliuzzi et al. successfully placed root analogue DLMF implants into patients. After 1-year follow-up, the implants were of good functional and aesthetic integration [145, 146]. Another 4-year follow-up research concerning DMLS mini-implant treatment in 62 patients was reported by Mangano's research group. The survival rate was 96.9%. The distance between the implant shoulder and the first visible bone-implant contact (DIB) was 0.38 mm for 1-year follow-up and 0.62 mm for 4-year follow-up [147].

In addition to the implants, the implant templates are widely processed by 3D printing. Mangano et al. used 3D-printed templates for guiding the denture implant in 20 patients partially edentulous. 96.4% of the templates were steady and suitable for clinical use [120]. Recently, Derksen et al. have conducted a prospective cohort study to evaluate the accuracy of 3D-printed templates in guiding implant position. Data comparisons were based on CBCT and intraoral scanning. The mean angular deviation was 2.72°, and the mean deviations at the implant's entry point and apex were, respectively, 0.75 mm and 1.06 mm. Multiple factors such as the implant's length and cortical interference may affect the accuracy [148].

#### 4.2.2. Transplantation

Transplantation, in terms of autotransplantation and allogenic tooth transplantation, is an old technique but is not widespread in dental clinics. With the development of AM technology, this old technique shows a new life. A custom-made implant drill was fabricated by the direct metal laser sintering 3D printing system. The allogenic tooth transplantation can be well-fitted in the recipient's alveolar bone using this 3D-printed drill. Periapical radiographs showed that the inflammatory and replacement resorption were stable at 4-month follow-up after the transplantation. This deemed low-cost modality inspires future researches concerning AM technology for tooth transplantation which reduces bone loss and improves the implant stability [149]. In a very recent case report, 3D-printed templates were also applied to autotransplantation with good clinical and radiologic results after 2-year follow-up [150]. Tooth transplantation could be an economic solution for patients by saving costs from an implant, abutment, and crown. In the future, more studies are inspired into this little researched field with the help of AM technology.

### 4.3. AM Technology in Prosthodontics

#### 4.3.1. Fixed Partial Dentures

**Figure 8:**
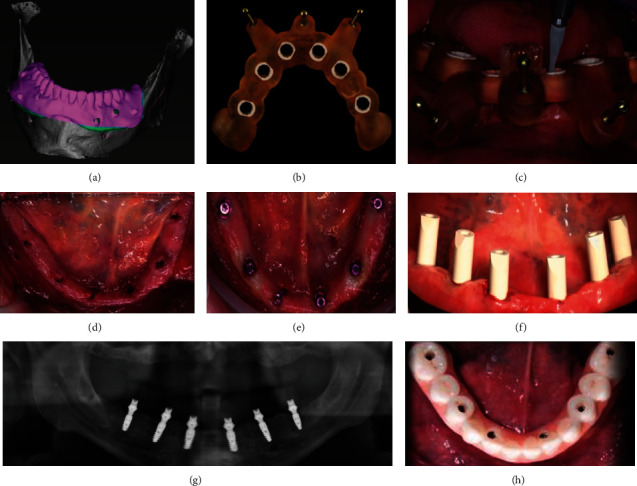
An implant-supported 3D-printed fixed denture was designed via CAD software (a). Meanwhile, a surgical stent was also printed (b) and used to guide the insertion of implants (c–f); the panoramic X-ray showed that the implants were well positioned (g); the printed fixed denture was subsequently delivered to the patient (h). Reprinted from [157].

Due to the complex and delicate anatomical structure in the oral cavity, the denture made by traditional impression methods and traditional restoration techniques is still inadequate. Dental digital impression technology combined with AM technology is expected to improve the accuracy of fixed restorations. Compared with the subtractive technique such as milling, the amount of materials used in AM is less, with almost no material loss [151]. AM can mainly be used to make personalized metal inner crowns, full crowns, interim crowns, and fixed bridges [152]. A single-unit crown by AM technology may be done in as little as approximately 20 minutes; the printed crown can be easily separated from the supports and rapidly cemented [153]. A good fitness is crucial to ensure the mechanical stability and health of surrounding soft tissue [154]. The fitness of the 3D-printed metal inner crown and the prepared tooth is significantly better than that of the traditional casting metal crown [119]. Alharbi et al. found that the marginal and internal gaps of 3D-printed interim restoration were lower than those of milled restorations [155]. By optimizing the printing parameters such as laser light intensity and printing orientation for each individual material, the accuracy of AM dental crowns would be greatly improved [153]. Taken together, the application of AM technology in fixed denture greatly simplifies the process and improves precision as well as material utilization (Figure 8) [119, 156, 157]. At present, Germany BEGO Company has already developed the compact DLP 3D printer to process commercialized permanent single crowns, crown bridges, inlays, onlays, and veneers (https://www.bego.com/3d-printing/).

#### 4.3.2. Removable Partial/Complete Denture

**Figure 9:**
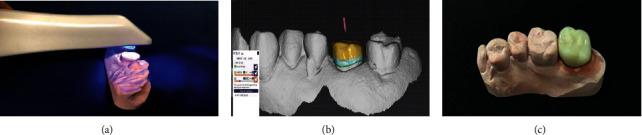
An intraoral optical scanner was used to scan the plaster model (a) to establish the digital model (b); then, the crown was designed and printed based on this digital model (c). Reprinted from [161].

The traditional removable partial/full denture design and fabrication commonly lead to pressure-induced mucosal pain and residual ridge resorption. Chen et al. [158] combined computer-aided optimization and additive manufacturing to process the jaw model and removable partial dentures. The optimized dentures were evenly attached to the mucosa; the uniformity was improved by 63%, and the contact pressure was decreased by 70%, thereby reducing pressure-induced mucosal pain and alveolar bone resorption. Due to the maturity of SLM technology, denture metal alloy stents can be processed to obtain a better fitness for clinical application [159]. The computer design system and AM technology can also be used to process resin-based denture bases and denture teeth (Figure 9) [160, 161]. Compared with the traditional compression molding technology, the AM technology generates lower volume and linear shrinkage of polymethylmethacrylate resin [162]. The adaptability of the AM denture base was better than that of the milling denture base in the maxilla, especially in the pressure-bearing areas [163, 164]. However, there are still some barriers to provide a well-fit RPD structure by AM and many factors affecting its mechanical properties. Noteworthy, material properties, AM procedure, and parameters used during manufacturing are influencing the mechanical properties of products [165]. Tregerman et al. reported that SLM Co-Cr alloy frames had better organization and mechanical properties when compared with the traditional cast or milled RPD frames [166]. On the contrary, Ye et al. found that the biggest misfits occurred in the SLM RPD frameworks [167]. Misfits in both studies were in the clinically acceptable range. Moreover, the reason for above discrepancy may be attributed to the difference between cast impression and intraoral scanning [166, 167]. Fabrication fitness may be affected by scanning accuracy, numerical control program, and the method to transform the data into a 3D model [168, 169]. In the future, more researches concerning the optimized parameters for AM denture should be carried out.

#### 4.3.3. Facial Prosthesis

The traditional prosthodontic reconstruction technique is difficult to accurately reproduce the complex defects, thus affecting the repair effect. At present, the AM technology is mainly used in the fabrication of the prosthesis support and the negative mold. The main materials used are metal powder, resin, resin wax, etc. However, the silicone materials for printing are unavailable for a long period. Recently, researchers successfully developed the directly printed silicone prosthesis [170]. In addition, Fripp Design Company developed a starch powder-based 3D system for printing medical-grade silicone [171]. Unkovskiy et al. applied directly printed silicone prostheses for a 40-year-old woman with a nasal defect. The interim prosthesis was acceptable; however, the position and marginal adaptation before definitive delivery of this prosthesis were difficult to evaluate [172]. Even so, this case report hints some cues in directly printed silicone prosthesis. Nuseir et al. compared a direct 3D printing workflow with the conventional workflow for a patient with a nasal defect. The total time required in 3D-printed nose prosthesis was 310 hours, compared with 500 hours in the conventional workflow [173]. The 3D printing workflow can lead to improved prosthesis reproducibility and aesthetic features and shows a great potential in treatment for patients with facial defects.

### 4.4. AM Technology in Orthodontics

#### 4.4.1. Digital Model

The diagnosis and treatment in orthodontics are commonly relying on plaster models. However, plaster models have high requirements on air humidity. If they are kept in places with high humidity, they are easily affected by moisture and deformed. Because of the low material strength, the plaster model is often damaged and loses its reference value in clinical practice. At present, resin models based on intraoral scanning and 3D printing technology have great advantages over plaster models in terms of accuracy, strength, and preservation [174].

#### 4.4.2. Personalized Brackets

In the beginning of the 21st century, German physician Wiechmann first introduced CAD/CAM and SLM technology to produce personalized lingual brackets [117, 118]. A recent preliminary clinical trial showed that the AM technique could also be used to fabricate customized esthetic ceramic brackets whose mechanical parameters were similar to those of commercial ceramic brackets [175]. These 3D-printed brackets could better fit the patient's tooth surface with a good aesthetic effect and overcome some drawbacks in traditional orthodontics such as high bracket loss rate, complex indirect redounding, and time-consuming manufacturing process [175].

#### 4.4.3. Clear Aligners

The application of clear aligners without brackets is in a rapid development stage; AM technology plays an important role in it. Briefly, the digital model of the dental jaw is reconstructed in three dimensions; then, the computer-aided diagnosis and design are used to simulate the movement of the teeth. After the plan is determined, the simulated dental jaw model is printed out using 3D printing technology; finally, the thermoforming technology is used to make an invisible appliance [176]. Currently, 3D printing is mainly used in the production of dentition models. The final invisible braces are still produced via traditional thermoforming technology. The printed appliance cannot be directly used in clinic due to the manufacturing accuracy, strength, and surface characteristics. A recent study showed that 3D-printed dental resin-based clear aligners were geometrically more accurate than thermoformed aligners. And the maximum load of 3D-printed cured dental aligners was 622 N for 2.93 mm displacement. These 3D-printed aligners were mechanically stronger than thermoformed aligners [177]. This shed light on the whole process of 3D printing in invisible orthodontics. Besides, Cassetta et al. had carried out an innovative orthodontic treatment method that combined computer-guided piezocision and clear aligners [178]. This combined technique reduced surgical time and patient discomfort, increased periodontal safety and patient acceptability, and achieved accurate control of orthodontic movement without the risk of losing anchorage. A 23-year-old woman with moderate crowding and a 13-year-old male patient with class II malocclusion have both been treated by this combined method. Treatment duration is greatly reduced to 6-8 months. Oral health-related quality of life and periodontal indexes are both improved after 2-year follow-up [179, 180]. However, the cases are limited and the follow-up time is not long; we need further investigation and practice to promote the use of this combined technique.

### 4.5. AM Technology in Endodontics (Table 4)

#### 4.5.1. Root Canal Therapy

The premise of perfect root canal treatment is to establish effective access to dental pulp cavity and root canal system. The 3D-printed templates can be widely used in localization of complex root canals. Fonseca Tavares et al. applied 3D-printed templates to access calcified central incisors [181]. Maia et al. used 3D-printed guides in accessing calcified canals of the maxillary premolar and first molar. After 15- or 30-day follow-up, all patients were asymptomatic [181, 182]. Lera-Mendes et al. applied this 3D-printed template to rapidly access the severely obliterated canals of maxillary second and third molars. After 3 months, the periapical tissue was greatly healed by the assessment of radiography [183].

Microguided endodontics is a recent accepted concept in root canal therapy which combines a small diameter bur (0.85 mm) with 3D-printed surgical templates. Connert et al. were the first to use this technique on mandibular incisors [184], and Torres et al. were the first to use this technique on maxillary incisors [185]. This novel technique minimizes invasion and apical extended access in incisors with canal calcification and apical periodontitis, while it shortens operation time on the patients [186]. However, microguided endodontics might not be currently used in the posterior region due to the space limitation. There needs further elaborate designs for 3D-printed templates and burs.

#### 4.5.2. Apical Surgery

Targeted endodontic microsurgery combined 3D-printed surgical guides with trephine burs can enhance the accuracy and efficacy of osteotomy and root-end resection, compared with traditional endodontic microsurgery [187]. Antal et al. applied SLA-fabricated surgical templates to resect 3 mm apical portion of the root in 11 patients with apical lesions. The mean apex removal and osteotomy depth error were 0.19 mm and 0.37 mm separately. No recurrence or complications were reported after 6-month follow-up [188]. Patients who were pathologically diagnosed as having a periapical cyst or granuloma were treated with precise osteotomy and root-end resection using a 3D-printed surgical guide. All patients were asymptomatic after 1-, 3-, or 6-month follow-up [189, 190]. Popowicz et al. reported the application of the 3D-printed polylactide surgical guide in 2 cases that underwent root-end resection. The two patients were asymptomatic at a 7- or 8-month follow-up visit. Radiographic examination showed complete healing with a radiodense area around the apex of the upper left second premolar. The cortical plate at the osteotomy site was restored to the original thickness [191]. This targeted endodontic microsurgery shows great benefit in challenging anatomic cases which involve fused molar roots, the palatal root of the maxillary first/second molar, roots of the mandibular first/second premolar adjacent to the mental nerve, and roots of mandibular molars with a thick buccal bone plate [190, 192]. Further studies with a larger group of patients are necessary to obtain landmark conclusions.

### 4.6. AM Technology in Periodontics (Table 5)

The periodontium is a complex tissue system consisting of several components like cementum, gingiva, and bone. The loss of periodontal tissue caused by periodontal disease is an irreversible process, and its regeneration has been a hot research topic in tissue engineering. The engineering of periodontal ligament (PDL), cementum, and the alveolar bone is based on a modular approach. Bioprinting using microfluidic AM technology could manufacture more highly intricate morphologies, internal structures, and architectures that accurately replicate the exact anatomical organization and biological function of periodontal tissues [122]. A solution containing keratinocytes and fibroblasts as ink components was successfully used to print out the epithelial cell rests of Malassez which are necessary for the initial stage of periodontal tissue formation [122]. Periodontal ligament stem cells (PDLSCs) show great potentials in periodontal tissue regeneration under the appropriate extracellular matrix (ECM) [193]. Gelatin methacrylate (GelMA) and poly (ethylene glycol) (PEG) dimethacrylate composition could serve as ECM materials. Cell and ECM interaction was screened by a cell-laden hydrogel array with the help of bioprinting. The cell viability and spreading area were decreased when the PEG ratio was increased [194].

Kim et al. used the compound ink of polycaprolactone (PCL) and hydroxyapatite (HA) as the raw material to align the teeth in vivo and in vitro. The normal anatomical structure of the body was restored, proving the possibility of using a dental scaffold to achieve tooth regeneration. Furthermore, cell-derived factor-1 (SDF1) and bone morphogenetic protein-7 (BMP7) on this porous scaffold can recruit endogenous cells with a homing effect which facilitates the generation of blood vessel-like, tooth-like, and periodontal tissues appearing at the interface between the stent and the alveolar bone [123]. Park et al. used a 3D bioprinting system to fabricate a PCL scaffold that efficiently promoted alveolar bone regeneration in a beagle defect model [195].

In clinical practice, Rasperini et al. were the first to report a personalized 3D-printed bioresorbable polymer scaffold for a 53-year-old male patient with periodontal defect. After 12-month follow-up, the patient gained a 3 mm clinical attachment of periodontal tissue and partial root coverage. However, at the 13-month follow-up visit, the scaffold was exposed and larger dehiscence was observed [196]. Although this case was unsuccessful in the long term, the approach gave some hints and experience for personalized oral tissue regeneration in clinical settings. Recently, Lei et al. applied a 3D-printed periodontal surgery template to guide tissue regeneration in a 36-year-old male patient with severe bone defects of the upper right lateral incisor. Advanced platelet-rich fibrin (A-PRF) and injected platelet-rich fibrin (I-PRF) from the patient's blood were mixed with Bio-Oss to form a 3D ideal shape. After a 15-month follow-up, the probing pocket depth was significantly reduced to a normal range. And the alveolar bone was regenerated at the treatment sit by the assessment of radiography [197].

Although, AM has been used extensively in guided tissue regeneration, the microlevel control of scaffold structure is limited by low resolution, material selection, and complexity [7]. Pilipchuk et al. introduced a novel strategy that combined 3D printing and micropatterning to advance the microlevel design of scaffolds. Results showed that the groove microdepth of the scaffold was a more important parameter than the width for promoting formation of cell alignment and increasing oriented collagen fiber density. This technique could efficiently achieve the formation of multiple tissues such as alveolar bone, cementum, and collagenous PDL-like tissue [198]. With the advancement of materials and biofabrication technologies, tissue engineering might progress into complex functional 3D organs in the future [199].

## 5. Conclusions and Challenges

AM technology is based on a digital model, layered scanning, and layer-by-layer stacking forming, and by stacking points, lines, surfaces, and bodies of layer materials, a nontraditional processing technology quickly produces three-dimensional objects. Compared with traditional technology, AM has some advantages. First, this technology dramatically reduces the duration of treatment. Secondly, the satisfaction degree and comfort level of patients are improved, and the patients can enjoy the convenience brought by personalized treatment and precision medicine. Thirdly, it has greatly improved the working efficiency of clinicians. Currently, it is widely used in maxillofacial surgery, denture implantation, prosthetics, orthodontics, endodontics, and periodontics [200–202].

With the increased clinical demand, it is imperative to transit printing from simple materials to specific biomaterials with physiological activities and functions [203]. In the future, AM should be more inclined to tissue regeneration, such as degradable biological scaffolds, reconstruction of tissue and organ structures, and permanent replacements in vivo. Despite the advantages of personalization and diversified printing materials, there are still some challenges in the development of AM technology.

1. The accuracy of 3D printing software, biomechanical properties of raw materials, and resolution of the 3D printer are crucial parameters that affect the quality of printed objects in the field of medicine and health care. Therefore, it is urgent to deepen the research on the manufacturing process and optimize the 3D software, materials, and equipment
2. The microprinter used in dental medicine can really realize in-house/chairside operation. However, the accuracy of 3D printing equipment as well as its intelligence needs to be further improved
3. The application of SLM technology to process removable partial denture is mature, but there is still insufficient research on the postprocessing technology that greatly impedes the large-scale application of SLM technology
4. In the application of tissue engineering scaffolds, the optimal degradation rate, mechanical properties, porosity, and pore size of bone tissue engineering scaffolds are still inconclusive [204]. There are few biodegradable materials applied in tissue engineering, and the current 3D printer resolution is in the micron level that has not reached the nanolevel of the jawbone. Therefore, it is necessary to increase the resolution of the 3D printer to improve the scaffold function
5. Due to the complex functions of tissues and organs, it may still take a long time to explore the cell sources and extracellular matrix types, as well as their interaction in the bioprinting [205]. Besides, the printing time will affect the cell activity. In order to speed up the printing, the printing pressure or energy intensity is commonly increased, but this will in turn damage the cells inside the stent, thereby resulting in impaired graft function [206]. The two facets should be weighed to make a reasonable choice. In addition to technical issues, bioprinting also has safety, ethics, and legal issues. These issues need to be considered during development
6. The expensive cost and high application threshold hinder further development of AM. Although the price of 3D printers has been gradually declining in recent years, 3D printers with good quality are still expensive. There also needs high investment in related supporting CT, MRI equipment, and computer-aided design software. In addition, the efficient use of equipment and software requires specialized technical training and multidisciplinary technical personnel division and cooperation
7. With the integration of multiple disciplines, AM technology will play a more important role in the diagnosis and treatment of dentistry diseases. Therefore, to establish a thorough and mature collaboration system is urgent
8. Emerging concept of 4D printing (3D plus time) [207] that accurately simulates the dynamic transformation of native tissues may remedy the shortcomings of 3D bioprinting. More researches are required to get new breakthrough in tissue regeneration using AM technology

## Figures and Tables

**Table 1:** Different types of AM technology in dentistry.

Technology	Company	Energy source	Raw material	Accuracy	Main property	Application
SLA [89, 96, 97]	3D systems (USA) Stratasys (USA) Formlabs (USA) DWS (Italy) Autodesk (USA)	Ultraviolet or visible light	Photosensitive resin, light curable liquid polymers, ceramic-filled resins, etc.	25-35 *μ*m	(i) High accuracy (ii) Rapid fabrication (iii) Need a support framework (iv) Brittle and easily broken (v) Strict storage	(i) Restoration of defected teeth (ii) Complete denture base (iii) Resin, dowel crown (iv) Orthodontic devices (aligners and retainers) (v) Surgical guide and splints (vi) Tooth root canal model (vii) Maxillofacial model
SLS/SLM [58, 91, 92, 98]	3D systems (USA) Blueprinter (Denmark) EOS (Germany)	High-power laser	Titanium and titanium alloy powder, cobalt chrome, aluminium, bronze alloy, stainless steel, nylon powder, elastomers, ceramics, etc.	20-50 *μ*m	(i) Good mechanical property (ii) Good accuracy (iii) High material utilization rate (iv) Prone to spherification (v) High cost (vi) Slow process	(i) Implant (ii) Alternative of large jawbone defects (iii) Removable partial denture (iv) Metal crown, coping, and bridges
EBM [92, 99–101]	Arcam (Sweden) FIT Group (Germany) Sciaky (USA)	Electron	Titanium and titanium alloy powder, cobalt base alloy powder, etc.	40-50 *μ*m	(i) Good accuracy (ii) Rapid fabrication (iii) High energy utilization rate (iv) High power density (v) Convenient focusing (vi) High cost (vii) Explosive risk	(i) Implant (ii) Fixation plate
FDM [93, 94, 102–105]	Stratasys (USA) MarkerBot (USA) RepRap (Germany) QiDi (China)	Extrusion	Thermoplastic filamentous material such as polylactic acid, polycarbonate, and PEEK	35-40 *μ*m	(i) Low- to midrange cost (ii) Relative accuracy (iii) Fast molding (iv) Good biocompatibility (v) Need a support	(i) Oral implant prosthesis (ii) Edentulous mandible (iii) Surgical guide (iv) Models (v) Simple anatomical parts
LOM [95, 106, 107]	3D systems (USA)	Laser	Thin material of metal and plastic	60-70 *μ*m	(i) Rapid fabrication (ii) Low cost (iii) Acceptable accuracy (iv) Low material utilization rate (v) Rough surface (vi) Easy to crack	(i) Jawbone model (ii) Denture models (iii) Surgical plan models
IJP [91, 108, 109]	3D systems (USA) Stratasys (USA)	Electric heater	Powder, living cells, biological materials, etc.	35-40 *μ*m	(i) High print speed (ii) Low cost (iii) Nozzle plugging	(i) Teeth (ii) Periodontal tissue (iii) Facial prosthesis

Notes: SLA: stereolithography; SLS: selective laser sintering; SLM: selective laser melting; DLMS: direct laser metal sintering; DLMF: direct laser metal formation; EBM: electron beam melting; FDM: fused deposition modeling; LOM: laminated object manufacturing; IJP: inkjet printing; PEEK: polyether ether ketone. DWS: Digital Wax Systems; EOS: Electro Optical Systems; FIT group: Fruth Innovative Technologien group; RepRap: replicating rapid prototype.

**Table 2:** Application of AM technology in maxillofacial surgery.

Author	Application	Cases	Scanning	Software	Material	Process	Main results
Melville et al. [127]	Surgery guide and fixation plate in maxillary reconstruction	1	MRI; CBCT	ProPlan (Materialise, USA)	Titanium; polyamide	SLS; FDM	Precisely correspond to the surgical defect Ideally restore the maxilla and midface Shorten operative time
Takano et al. [128]	Maxillary reconstruction	1	CT	Mimics (Materialize, Belgium)	Titanium	3D printing (Stratasys, USA)	Improve safety Shorten surgery time Achieve good function and esthetic outcomes
Abo Sharkh et al. [129]	Cutting guides and jaw models in maxillofacial reconstruction	19	CT	3DSlicer (NIH, USA) Meshmixer (Autodesk, USA)	Resin	SLA (Formlabs, USA) FDM (QiDi, China)	The average time for VSP and fabrication of cutting guides was 158 minutes The average cost was $18.01 Canadian dollars
Shaheen et al. [132]	Occlusal splints in orthognathic surgery	20	CT (Siemens, Germany) Optical scanner (SmartOptics, Germany) CBCT (Planmeca, Finland)	ProPlan (Materialise, Belgium) 3-matic (Materialise, Belgium)	Biocompatible material (MED610)	SLA (Objet Connex 350, Stratasys, USA)	The mean absolute distance error was 0.4 mm
Dumrongwongsiri et al. [133]	Spacers in orthognathic surgery	12	CBCT	3-matic (Materialise, Belgium)	Biocompatible material (MED610)	3D printing (Stratasys, USA)	Mean preoperative visual analogue scale score was improved by 47.82% Mean facial surface area discrepancy index was corrected by 3.16%
Zhang et al. [134]	Mandibular models and surgical templates in orthognathic surgery and mandibular contour osteoplasty	10	CBCT	Mimics (Materialise, Belgium)	Plastic	3D printing (Objet Eden 250, Israel)	The right gonial angle was improved from 128.20° to 120.35° The left gonial angle was improved from 129.91° to 120.74°
Shaheen et al. [135]	Splints in bimaxillary orthognathic surgery	20	CT (Siemens, Germany) CBCT (Planmeca, Finland) Intraoral scan (3Shape, Denmark)	ProPlan (Materialise, Belgium) 3-matic (Materialise, Belgium)	Biocompatible material	3D printing (Objet Connex 350, Stratasys, USA)	95% of 3D-printed splints were clinically accepted
Heufelder et al. [136]	Surgical guides and implants in bimaxillary orthognathic surgery	22	CT (Siemens, Germany) Optical scan	ProPlan (Materialise, Belgium)	Not mentioned	SLM	The median deviation of the maxilla position was 0.39 mm between the preoperative plan and surgical result The accuracy of left-right, up-down, and anterior-posterior positioning was 0.3 mm,0.33 mm, and 0.7mm, respectively
Li et al. [137]	Cutting guides and fixation plates in bimaxillary orthognathic surgery	10	CT (GE Healthcare, USA) Optical scan (SmartOptics AS, Germany)	ProPlan (Materialise, Belgium) Geomagic Studio (Geomagic, USA)	Ti6Al4V; photosensitive resin	EBM (Arcam AB, Sweden) SLS (3D system, USA)	Achieve good final occlusion The median surgical time was 160 minutes Postoperative nerve parenthesis was recovered with 2-3 months

Note: cases: the number of patients enrolled in the research; MRI: magnetic resonance imaging; CT: computed tomography; CBCT: cone-beam computed tomography; NIH: National Institutes of Health; VSP: virtual surgical planning; SLS: selective laser sintering; SLM: selective laser melting; SLA: stereolithography; FDM: fused deposition modeling; EBM: electron beam melting.

**Table 3:** Application of AM technology in denture implantation.

Author	Application	Cases	Specification	Scanning	Software	Material	Process	Main results
Tunchel et al. [121]	Dental implant	82	Tixos^R^ (Leader Implants, Italy) 3.3 mm/3.75 mm/4.5 mm	CBCT	Not mentioned	Ti-6Al-4V alloy	DLMF (EosyntM270, Germany)	3-year follow-up: survival rate (94.5%), success rate (94.3%) DIB (0.75 for 1 year; 0.89 for 3 years)
Mangano et al. [145]	Immediate dental implant	15	Root analogue	CBCT (CS9300, USA)	Mimics (Materialise, Belgium) Magics (Materialise, Belgium) PTC Group (Needham, USA)	Ti-6Al-4V alloy	DLMS (Leader Implants, Italy)	1-year follow-up: survival rate (100%) DIB (0.7 mm)
Figliuzzi et al. [146]	Immediate dental implant in the anterior maxilla	1	Root analogue	CBCT (CS9300, USA)	Mimics (Materialise, Belgium) Magics (Materialise, Belgium) PTC group (Needham, USA)	Ti-6Al-4V alloy	DLMS	1-year follow-up: good functional and aesthetic integration
Mangano et al. [147]	Immediate loading of four unsplinted implants	62	Tixos^R^ (Leader Implants, Italy) 2.7 mm/3.2 mm	CBCT (CS9300, USA)	Mimics (Materialise, Belgium)	Ti-6Al-4V alloy	DLMS (EOSINT M270, Germany)	4-year follow-up: survival rate (96.9%) DIB (0.38 mm for 1 year; 0.62 mm for 4 years) Biological complications (6%), prosthetic complications (12.9%)
Mangano et al. [120]	Implant templates	20	Tooth-supported	CBCT (CS9300, USA)	Nauta (DWS, Vicenza)	Resin	SLA (XFAB2000, DWS, Vicenza)	96.4% of the templates were steady and suitable for clinical use
Derksen et al. [148]	Implant templates	66	Tooth-supported	CBCT (Morita, Japan)	coDiagnostiX (Dental Wings, Canada)	Biomaterial (MED 610)	3D printing (Eden 260V, Stratasys, USA)	The mean angular deviation was 2.72° The mean deviations at the implant's entry point and apex were 0.75 mm and 1.06 mm, respectively
Xu et al. [149]	Implant drills in allogenic tooth transplantation	1	Not mentioned	CBCT	Not mentioned	Metal powder	DLMS	The donor tooth fitted well in the recipient's alveolar bone The inflammatory and replacement resorption was stable after 4 months
Mena-Álvarez et al. [150]	Autotransplantation templates	1	Tooth-supported	CBCT (White Fox, France)	Not mentioned	Not mentioned	3D printing (Explora 3D lab, Spain)	2-year follow-up: accurate placement of the donor tooth in the recipient site with good physiological clinical and radiologic results

Note: cases: the number of patients enrolled in the research; DIB: distance between the implant shoulder and the first visible bone-implant contact; CBCT: cone-beam computed tomography; Ti-6Al-4V: titanium-6 aluminium-4 vanadium; SLA: stereolithography; DLMS: direct laser metal sintering; DLMF: direct laser metal formation.

**Table 4:** Application of AM technology in endodontics.

Author	Application	Cases	Scanning	Software	Material	Process	Main results
Fonseca Tavares et al. [181]	Guided root canal access	2	CBCT	Simplant (Materialise, Belgium)	Not mentioned	3D printing (Objet Eden 260v, Stratasys, USA)	The calcified root canals of central incisors were successfully accessed After 15- or 30-day follow-up, the two patients were asymptomatic
Maia et al. [182]	Guided root canal access	3	CBCT (i-CAT Classic, Brazil) Intraoral scanner (3Shape, Denmark)	coDiagnostiX (Dental Wings GmbH, Germany) Simplant (Materialise, Belgium)	Not mentioned	3D printing (Objet Eden260v, Stratasys, USA)	The calcified root canals were successfully accessed After 12 months, the periapical tissue was completely healed
Lara-Mendes et al. [183]	Guided root canal access	1	CBCT (i-CAT, PA)	Simplant (Materialise, Belgium)	Not mentioned	3D printing (Objet Eden 260v, USA)	The calcified root canals of tooth 27 and 28 were rapidly accessed After 3 months, the periapical lesions were reduced
Connert et al. [184]	Microguided endodontics	1	CBCT (Accuitomo 80, USA)	coDiagnostiX^TM^ (Dental Wings Inc., Canada)	Note mentioned	3D printing (Objet Eden260v, Stratasys, USA)	The obliterated root canal of tooth 31 and 41 was precisely accessed
Torres et al. [185]	Microguided endodontics	1	CBCT (New Tom VGi evo, Italy)	3-matic (Materialise, Belgium)	Biocompatible material (MED 610)	3D printing (Objet Connex 350, Stratasys, USA)	The obliterated root canal of tooth 22 was precisely accessed The apical area was completely healed after 6-month follow-up
Antal et al. [188]	Root-end resection guide	11	CBCT (i-CAT Next Generation, USA)	SMARTGuide (DicomLAB Dental, Hungary)	Metal	SLA (ProJet MD 3510, 3D system, USA)	The median angular deviation was 3.95° The mean apex removal and osteotomy depth error were 0.19 mm and 0.37 mm separately
Ye et al. [189]	Periapical surgery guide	1	CBCT (i-CAT 17-19, USA)	Simplant (Materialise, Belgium)	Not mentioned	3D printing (3510SD, 3D system, USA)	The root ends were resected accurately The patient was asymptomatic after 6 months
Giacomino et al. [190]	Osteotomy and root-end resection guide	3	CBCT (3D Accuitomo 170, CA)	Mimics (Materialise, Belgium) Blue Sky Plan (Blue Sky Bio, USA)	Not mentioned	3D printing (Objet 260 Connex 3, USA)	All patients were asymptomatic after 1 month or 3 months
Popowicz et al. [191]	Root-end resection guide	2	CBCT (CS8100, Carestream Dental, USA)	DDS-Pro (Natrodent Polska, Poland)	Polylactide	3D printing (Prusa i3 MK2S, Czech)	The patients were asymptomatic at a 7- or 8-month follow-up visit Radiologic assessment showed complete healing with a radiodense area around the apex
Ahn et al. [192]	Periapical surgery guide	1	CBCT (Alphrad 3030, Japan)	OnDemand3D (Cybermed, Korea)	Biocompatible clear resin	3D printing (Object Eden 260v, Stratasys, USA)	No postoperative complications were reported

Notes: cases: the number of patients enrolled in the research; CBCT: cone-beam computed tomography; SLA: stereolithography.

**Table 5:** Application of AM technology in periodontics.

Author	Application	Cases	Scanning	Software	Material	Process	Main results
Kim et al. [123]	Guided tissue regeneration (In vivo)	Not applicable	Laser scanning	Not mentioned	PCL, HA	3D printing	After 9 weeks, a putative periodontal ligament and native alveolar bone were regenerated at the interface incisor scaffold
Park et al. [195]	Scaffold for alveolar bone regeneration (in vivo)	Not applicable	CT	Not mentioned	PCL	3D bioprinting system (laboratory-made system in Korea Institute of Machinery and Materials, Korea)	New bone was formed adjacent to the scaffold PCL blocks with 400/1200 lattices were inclined to more new bone formation
Rasperini et al. [196]	Scaffold for periodontal repair	1	CT	NX 7.5 (Siemens PLM Software, USA) Mimics (Materialise, USA)	PCL	SLS (Formiga P100 System; EOS, Germany)	After 12-month follow-up, the patient gained a 3 mm clinical attachment and partial root coverage After 13-month follow-up, the scaffold was exposed
Lei et al. [197]	Guided tissue regeneration	1	CBCT	Mimics (Materialise, Belgium)	Biocompatible material (MED 610)	PolyJet (Objet Connex 350, Stratasys, USA)	After 3 months, the probing pocket depth was greatly reduced After 6 months, bone was regenerated by the assessment of radiography
Pilipchuk et al. [198]	Scaffold for dentin, ligament, and bone regeneration (in vitro & in vivo)	Not applicable	Not mentioned	NX 7.5 (Siemens PLM Software, USA)	PCL, HA	SLS	Groove microdepth was a more important parameter than width for promoting formation of cell alignment and increasing oriented collagen fiber density

Notes: CT: computed tomography; CBCT: cone-beam computed tomography; PCL: polycaprolactone; HA: hydroxyapatite; SLS: selective laser sintering.

## Data Availability

No data were used to support this study.
